# A case of perforated Meckel’s diverticulitis tethered to the umbilicus associated with a urachal remnant

**DOI:** 10.1093/jscr/rjad158

**Published:** 2023-04-06

**Authors:** Christopher Woods, Thomas Whitehead-Clarke, Benita Stevenson, Nirav Patel, Myutan Kulendran

**Affiliations:** General Surgery, St George’s Hospital, London, UK; Centre for 3D models of Health and disease, University College London, London, UK; Pathology, St George’s Hospital, London, UK; Radiology, St George’s Hospital, London, UK; General Surgery, St George’s Hospital, London, UK

**Keywords:** Meckels diverticulitis, urachal remnant, recurrent UTI, chronic abdominal pain

## Abstract

Meckel’s diverticulum (MD) occurs in 2% of the population and is often asymptomatic. It is an embryological remnant of the oomphalomesenteric duct and can be associated with another embryonic structure—the urachus. A 23-year-old male presented with generalized abdominal pain and fever on a background of chronic abdominal pain and recurrent urinary infections. A CT scan of the abdomen and pelvis revealed an inflamed MD. Next day, the patient deteriorated and was taken to theatre. The MD was found to be both perforated and tethered to the umbilicus, which itself was directly related to an abnormal extra-peritoneal structure—shown to be a urachal remnant. Such cases pose diagnostic and therapeutic challenges. Young males with chronic abdominal pain and recurrent urinary infections should be thoroughly investigated for such pathology. Laparoscopic approach to such cases should be undertaken with caution due to possible umbilical tethering.

## INTRODUCTION

Meckel’s diverticulum (MD) is a congenital abnormality of the small intestine caused by incomplete atrophy of the oomphalomesenteric duct. Some reviews describe an incidence of around 0.3–2.9% [1], with other autopsy studies suggesting an incidence of 1.2% [2]. The majority of MD cases are asymptomatic, with symptomatic patients making up between 4.2 [2] and 9% [3] of cases. Symptomatic patients are 1.5–4 times more likely to be male than female [1]. Common symptoms include intestinal obstruction, hemorrhage and inflammation [1].

The urachus is an embryological ductal remnant that connects the anterior dome of the bladder to the umbilicus, and is formed from the cloaca and the allantois [4]. During gestation, the bladder descends caudally, stretching the urachus and causing its obliteration. Failure of this process leads to a number of urachal abnormalities. A lack of epidemiological reports means that the incidence of urachal anomalies is not well understood [4]; however, some estimate an incidence of around 1% of the pediatric population [5].

Both MD and urachal remnants are congenital abnormalities of embryological tracts that pass through the umbilicus. Cases of both abnormalities occurring simultaneously are uncommon but have been documented [6, 7].

## CASE DESCRIPTION

A 23-year-old male presented with a six-day history of worsening supraumbilical abdominal pain associated with fevers and vomiting. His white blood cell count was 17.7 and his C-reactive protein was 25 with otherwise normal blood results. On examination, his abdomen was soft with tenderness and guarding periumbilically.

The patient’s medical history included several years’ of chronic intermittent abdominal pain. Painful episodes usually lasted between one and three weeks, prompting several visits to hospital. On each occasion, his blood tests were normal and he was discharged home without imaging. To investigate this pain, the patient underwent outpatient flexible sigmoidoscopy as well as abdominal ultrasound scan results of both were normal. During this time, the patient also underwent a US scan of his urinary tract to investigate recurrent urinary tract infections (UTI). This scan was also normal.

Given his severe pain and raised inflammatory markers, a CT scan of the abdomen and pelvis was organized, revealing an inflamed MD with associated free fluid within the pelvis. The patient was admitted and treated conservatively with antibiotics.

**Figure 1:**
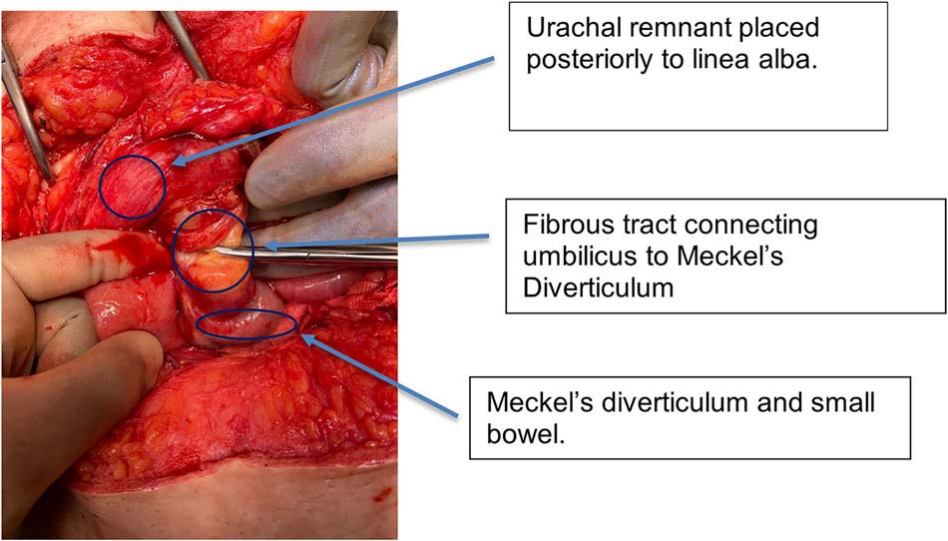
Intra-operative photograph showing small bowel and MD adherent to umbilicus.

**Figure 2:**
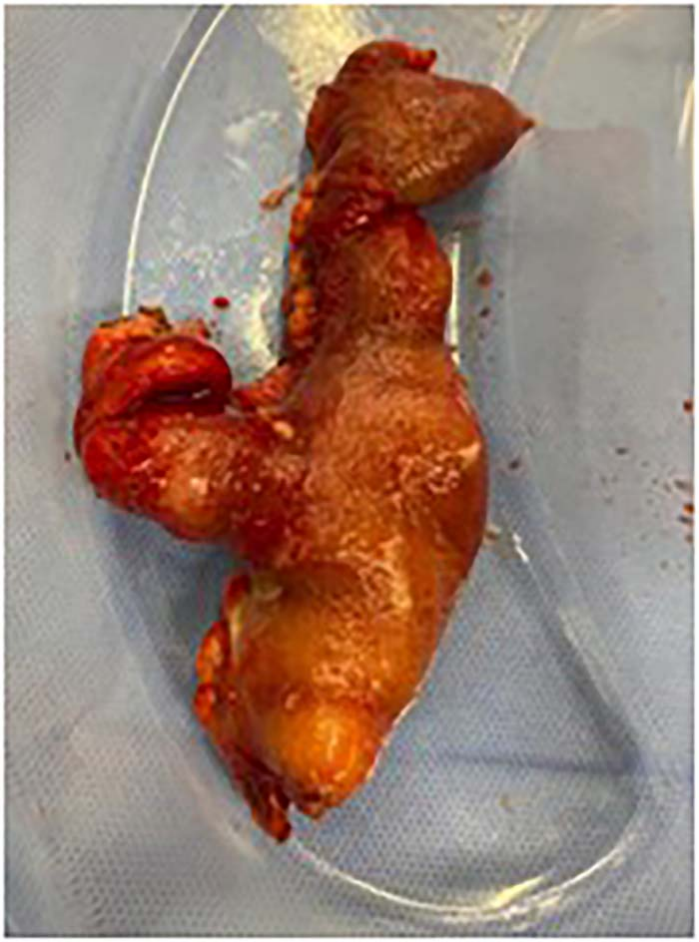
Resected specimen—small bowel with MD—resected from adherent umbilicus.

The following day, the patient deteriorated, suffering persistent tachycardia, hypotension and fevers and so was taken to theatre. In theatre, surgeons performed a midline laparotomy and identified an inflamed MD that had now perforated causing four-quadrant purulent peritonitis. The MD was found directly in the midline, tethered to the umbilicus (Fig. 1). Surgeons also noted a thickened, abnormal preperitoneal midline structure continuous with the umbilicus. The fibrous tissue tethering the MD to the umbilicus was divided, and the affected bowel was resected (Fig. 2) with a stapled side-to-side anastomosis. The unusual preperitoneal tissue was resected and sent for histology. The patient’s post-operative period was complicated by small intra-abdominal collections, which were managed conservatively with antibiotics. The patient was discharged eleven days after admission.

**Figure 3:**
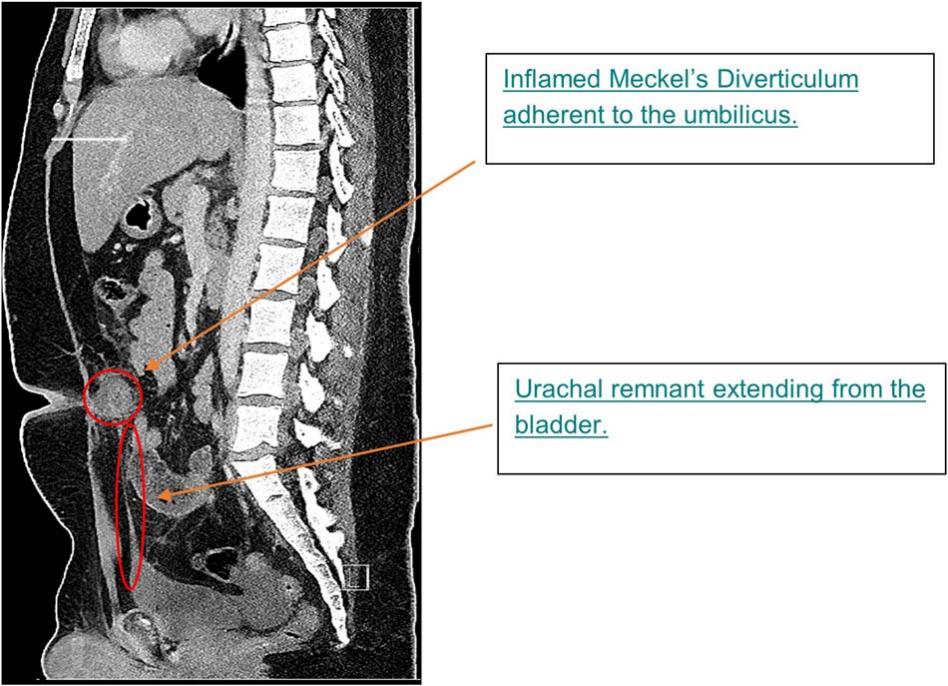
Sagittal image from CTAP at admission; CT showing inflamed MD at level of umbilicus and urachal remnant at apex of bladder tracking up to inflammatory mass.

Histopathological assessment of the preperitoneal tissue showed heterotopic gastric tissue with a surrounding smooth muscle layer, previously described in other omphalomesenteric duct remnants [8]. Detailed review of the patient’s pre-operative CT scan revealed a midline urachal remnant (Fig. 3), associated with tenting of the urinary bladder, consistent with a likely vesicourachal diverticulum.

**Figure 4:**
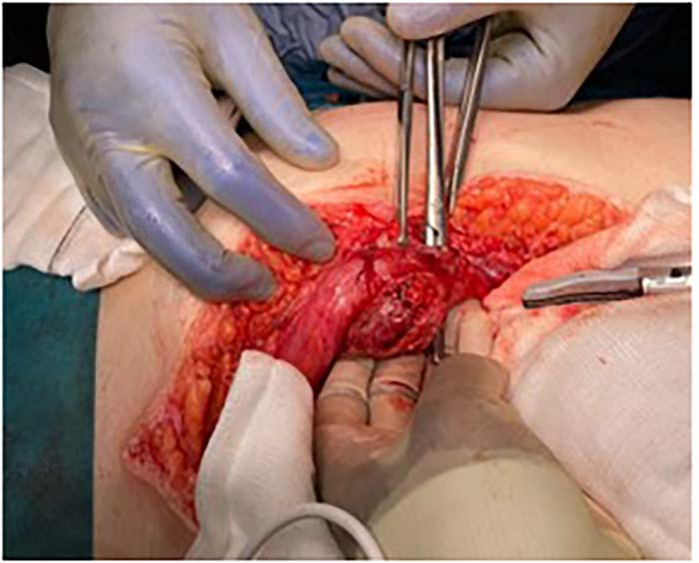
Umbilicus and abdominal wall following resection of MD.

The patient was reviewed as an outpatient one week following his discharge. The patient had no ongoing clinical issues and was discharged with referral to a urology outpatient clinic.

## DISCUSSION

We describe a rare case of simultaneous perforated MD tethered at the umbilicus with associated urachal remnant. From histological and radiological evidence, it is likely the patient suffered from a vesicourachal diverticulum, one of five different congenital urachal abnormalities, including a patent urachus, umbilical-urachal sinus, urachal cyst and alternating sinus [4]. Possible complications of vesicourachal diverticuli include spontaneous or traumatic rupture, recurrent infection (as with our patient) and stone formation [4]. Vesicourachal diverticuli tend to present clinical issues for adults rather than children [4], and diagnosis can be made by CT imaging as well as a voiding cystourethrogram and cystoscopy [4].

Whilst this case is rare, similar presentations have been recorded in the literature. One such case describes a one year-old boy with an MD tethered to the umbilicus and simultaneous patent urachus [7]. The earliest case identifying both pathologies simultaneously was published in 1968—where a patient suffering long-term umbilical discharge from a urachal remnant was found to have an untethered MD during surgery [6]. A case similar to ours with a tethered midline MD was reported in a pediatric patient in 2005, where at the time, pain and peritonism was initially confused for acute appendicitis [9]. Tethering of MD to the umbilicus has also been demonstrated among case series of CT images [10].

Elective surgical management of both MD and urachal anomalies are issues for debate. For MD, around 4% of patients will require hospital admission for symptoms and 2.9% will go on to require surgery [2]. The current mortality is estimated at around 0.02%, and the historical mortality at around 0.01%. Some have therefore argued that elective resection of asymptomatic MD is inappropriate given the number needed to resect in order to prevent death [2].

There is no established protocol for the management of asymptomatic urachal anomalies [4]. Symptomatic urachal remnants can be treated conservatively in children, where they often resolve within the first year of life [4]. For older patients, wide local excision may help prevent future malignancy, but some believe the number needed to treat for such an indication is too high [5].

Laparoscopic excision of urachal anomalies is now widely practiced [11, 12]. One series of 14 patients by Siow et al. reports successful laparoscopic wide local excision and ligation of the urachal remnant close to the umbilicus using ligation clips [12].

This unique case provides interesting clinical lessons for managing similar patients. Clinicians should be particularly mindful of the necessary investigations for young men with recurrent urinary infections as well as chronic abdominal pain. When considering a surgical approach to treat MD, surgeons should consider the possibility of umbilical tethering before proceeding laparoscopically and consider avoiding umbilical trocar placement.
